# Statistical hypothesis testing of factor loading in principal component analysis and its application to metabolite set enrichment analysis

**DOI:** 10.1186/1471-2105-15-51

**Published:** 2014-02-21

**Authors:** Hiroyuki Yamamoto, Tamaki Fujimori, Hajime Sato, Gen Ishikawa, Kenjiro Kami, Yoshiaki Ohashi

**Affiliations:** 1 Human Metabolome Technologies, Inc, 246-2 Mizukami, Kakuganji, Tsuruoka, Yamagata 997-0052, Japan

**Keywords:** Principal component analysis, Statistical hypothesis testing of factor loading, Metabolite set enrichment analysis

## Abstract

**Background:**

Principal component analysis (PCA) has been widely used to visualize high-dimensional metabolomic data in a two- or three-dimensional subspace. In metabolomics, some metabolites (e.g., the top 10 metabolites) have been subjectively selected when using factor loading in PCA, and biological inferences are made for these metabolites. However, this approach may lead to biased biological inferences because these metabolites are not objectively selected with statistical criteria.

**Results:**

We propose a statistical procedure that selects metabolites with statistical hypothesis testing of the factor loading in PCA and makes biological inferences about these significant metabolites with a metabolite set enrichment analysis (MSEA). This procedure depends on the fact that the eigenvector in PCA for autoscaled data is proportional to the correlation coefficient between the PC score and each metabolite level. We applied this approach to two sets of metabolomic data from mouse liver samples: 136 of 282 metabolites in the first case study and 66 of 275 metabolites in the second case study were statistically significant. This result suggests that to set the number of metabolites before the analysis is inappropriate because the number of significant metabolites differs in each study when factor loading is used in PCA. Moreover, when an MSEA of these significant metabolites was performed, significant metabolic pathways were detected, which were acceptable in terms of previous biological knowledge.

**Conclusions:**

It is essential to select metabolites statistically to make unbiased biological inferences from metabolomic data when using factor loading in PCA. We propose a statistical procedure to select metabolites with statistical hypothesis testing of the factor loading in PCA, and to draw biological inferences about these significant metabolites with MSEA. We have developed an R package “mseapca” to facilitate this approach. The “mseapca” package is publicly available at the CRAN website.

## Background

Metabolomics is a science based on the exhaustive profiling of metabolites. Various analytical technologies are used in metabolomic research, including capillary electrophoresis–mass spectrometry (CE–MS), liquid chromatography–MS, gas chromatography–MS, and nuclear magnetic resonance. The statistical analysis of the analytical data obtained has been studied in chemometrics research [1]. Chemometric approaches that commence with a multivariate analysis, such as principal component analysis (PCA) [2], partial least squares [3], canonical correlation analysis [4], and so on, have been predominantly applied in metabolomics.

PCA [2] is routinely used to visualize high-dimensional metabolomic data in a two- or three-dimensional subspace. A scatter plot of PC score vectors (a “scores plot”) can be used to detect outliers or to identify biologically interpretable patterns. Typically, when a specific PC score is found to be related to a phenotype of interest [5,6], such as a time course or group information, the corresponding factor loading is evaluated to discern meaningful metabolites from which to draw biological inferences.

In many metabolomic research articles [7-9], an eigenvector in PCA (Eq. 1–1) has been used as the factor loading. To draw biological inferences, some metabolites (e.g., the top 10 metabolites) are subjectively selected using the eigenvector. However, this approach has several problems. For example, many metabolites may vary with phenotype in one study, whereas only a few metabolites vary with phenotype in another study. With the existing approach, which uses the eigenvector, this is equivalent to using the same number of metabolites to draw biological inferences from these different studies. Consequently, biological interpretations might be made using an insignificant metabolite that varies irrelevantly with phenotype.

The eigenvectors for autoscaled data in PCA [10] are proportional to the correlation coefficients between the PC scores and the variables. This fact is well established in the multivariate analysis literature [11], but does not appear to be appreciated in metabolomic analyses. In the present study, “factor loading” is defined as the correlation coefficients between the PC scores and the variables. This definition can be used to perform statistical hypothesis testing and to select significant metabolites objectively using statistical criteria. The significance of factor loading in PCA can also be computed with a resampling approach, such as bootstrapping, although this is not exact [12].

Significant metabolites are selected according to the significance of factor loading or other methods of variable selection in supervised learning approaches, such as support vector machine, random forest, and so on, and then biological inferences are drawn for these metabolites by biologists. Biologists often draw these inferences with respect to a biological functional unit, such as a metabolic pathway (e.g., “glycolysis is notably activated” or “amino acid metabolism is significantly suppressed”). In gene expression analyses, gene set enrichment analysis (GSEA) has been used to identify significant gene sets using gene ontology (GO) terms. In metabolomics, metabolite set enrichment analysis (MSEA) [13,14] can be used to identify significant metabolic pathways. MSEA has been computed with several approaches, including overrepresentation analysis (ORA) [15], Subramanian’s GSEA [16], and the global test [17]. MSEA is a convenient method for drawing biological inferences from metabolomic data, but this approach has not been applied to metabolites selected by factor loading in PCA. Recently, web tools for MSEA have been developed [13,14]. However, no tools that can perform our workflow, including the statistical hypothesis testing of factor loading in PCA, have existed in a single platform. In the present study, we performed statistical hypothesis testing of the factor loading in PCA for two metabolomic datasets from mouse liver samples as case studies. This approach can be used to select significant metabolites when using factor loading in PCA, and MSEA with an ORA approach can be applied to these significant metabolites. We developed the R package “mseapca” to compute the sequence from the statistical hypothesis testing of factor loading in PCA to MSEA.

## Methods

### Statistical hypothesis testing of factor loading in PCA

Consider a mean-centered data matrix **X** that has samples in each row and variables in each column. The score vector is related to the data matrix by **t** = **Xw**, where **w** is a vector of weights. PCA is formulated as the optimization problem of maximizing the variance of the score vector **t**:

(1-1) $$\begin{array}{c} \mathrm{maxvar}\left(t\right)\\ \mathrm{subject}\;\mathrm{to}\;w^{\prime }w=1 \end{array}$$

and the weight vector **w** is often used for factor loading. After transformation, eq. (1–1) can be rewritten as the eigenvalue problem:

(1-2) $$\frac{1}{n\;-\;1}X^{\prime }\mathrm{Xw}\;=\;\lambda w$$

The eigenvector **w** and eigenvalue λ of eq. (1–2) can be computed using numerical computation libraries for singular value decomposition. The eigenvalue λ corresponds to the variance of the PC score vector formed using the associated eigenvector as the weight vector.

The coefficient of the correlation between the PC score and the p-th variable can be defined as:

(1-3) $$\mathrm{corr}\left(t,x_{p}\right)=\frac{t^{\prime }x_{p}/n-1}{\sqrt{\mathrm{var}\left(t\right)}\sqrt{\mathrm{var}\left(x_{p}\right)}}$$

where **t**' is the transpose of **t**. Introducing **c** as the column vector in which the p-th element is 1 and the others are 0, so that **x**_**p**_ = **Xc**, we have:

(1-4) $$\mathrm{corr}\left(t,x_{p}\right)=\frac{w^{\prime }X^{\prime }\mathrm{Xc}/n‒1}{\sqrt{\mathrm{var}\left(t\right)}\sqrt{\mathrm{var}\left(x_{p}\right)}}$$

Transposing eq. (1–2) gives **w**′**X**′**X**/*n* - 1 = *λ***w** ', which can be substituted in eq. (1–4), giving:

(1-5) $$\mathrm{corr}\left(t,x_{p}\right)\;=\;\frac{\lambda w^{\prime }c}{\sqrt{\mathrm{var}\left(t\right)}\sqrt{\mathrm{var}\left(x_{p}\right)}}$$

The variance of the score vector can then be replaced with λ and the standard deviation of **x**_**p**_ is replaced with σ_p_. Finally, the correlation between the PC score and the variables can be written as:

(1-6) $$\mathrm{corr}\left(t,x_{p}\right)\;=\;\frac{\lambda w^{\prime }c}{\sqrt{\mathrm{var}\left(t\right)}\sqrt{\mathrm{var}\left(x_{p}\right)}}\;=\;\frac{\lambda w_{p}}{\sqrt{\lambda \sigma_{p}}}\;=\;\frac{\sqrt{\lambda w_{p}}}{\sigma_{p}}$$

With data scaled to unit variance (autoscaling), the weight **w**_p_ is proportional to the correlation coefficient between the PC score and variable **x**_**p**_ because σ_p_ = 1 in eq. (1–6). Thus, the factor loading can be defined as the correlation coefficient in eq. (1–6). On the basis of this definition, we can perform a statistical test for factor loading in PCA, using the well-known fact that for a correlation coefficient r, the statistic:

(1-7) $$t=\frac{r\sqrt{n-2}}{\sqrt{1-r^{2}}}$$

has a *t*-distribution with (n – 2) degrees of freedom. We can then select variables that have a statistically significant correlation to the PC score and draw biological inferences using these variables.

### Sample preparation, metabolomic analysis, and data processing

BKS.Cg-m+/m+/Jcl (normal) mice, 12 h-fasted normal mice, BKS.C - +Lepr^db^/+Lepr^db^/Jcl (*db/db*) mice, and *db/db* mice orally administered pioglitazone for 10 days were used. The mice were 7-week-old males, and were given unlimited access to food and water, except those on the 12 h fast. The concentration of pioglitazone administered was 100 mg/10 mL per kg. The pioglitazone was purchased from Takeda Pharmaceutical Co. Ltd (Doshomachi, Osaka, Japan), and was purified by the NARD Institute Ltd (Amagasaki, Hyogo, Japan). After sampling, the livers were excised and stored at -80°C. All experiments, from the purchase and breeding of the mice to the collection of their liver samples, were performed at Kitayama Labes Co. Ltd (Ina, Nagano, Japan). The sample preparation procedure used to extract the metabolites has been described by Ooga et al. [18].

The metabolite extracts were measured with CE–time-of-flight MS (CE–TOFMS) using the Agilent Capillary Electrophoresis System equipped with an Agilent 6210 time-of-flight mass spectrometer, an Agilent 1100 isocratic high-performance liquid chromatography pump, an Agilent G1603A CE–MS adapter kit, and an Agilent G1607A CE–ESI–MS Sprayer Kit (Agilent Technologies, Waldbronn, Germany). The system was controlled with the G2201AA ChemStation Software version B.03.01 for CE (Agilent Technologies). Modified analytical methods for the measurement of cationic [19] and anionic metabolites [20] were used. The measurement data were processed with peak processing software [21]. The signal peaks corresponding to isotopomers, adduct ions, and other product ions of known metabolites were excluded. All signal peaks potentially corresponding to authentic compounds were then extracted, and their migration times (MTs) were normalized using those of the internal standards (methionine sulfone and d-camphor-10-sulfonic acid for cations and anions, respectively). The peaks were then aligned according to their m/z values and normalized MT values. Finally, the peak areas were normalized against those of the internal standards. The resultant relative area values were further normalized by the sample weight. Annotation tables were produced from the CE–TOFMS measurements of standard compounds, and were aligned with the datasets according to their similar m/z values and normalized MT values.

### Statistical analysis

In this study, all computations were performed with R [22] and the “mseapca” [23] package. A value of 0 was imputed to missing values for the computation of PCA. A metabolite set list was created with reference to the Kyoto Encyclopedia of Genes and Genomes (KEGG) [24], which was partly modified by manual curation. The xml file of the metabolite set list used in this study is included in the “mseapca” package.

### Software

**Figure 1:**
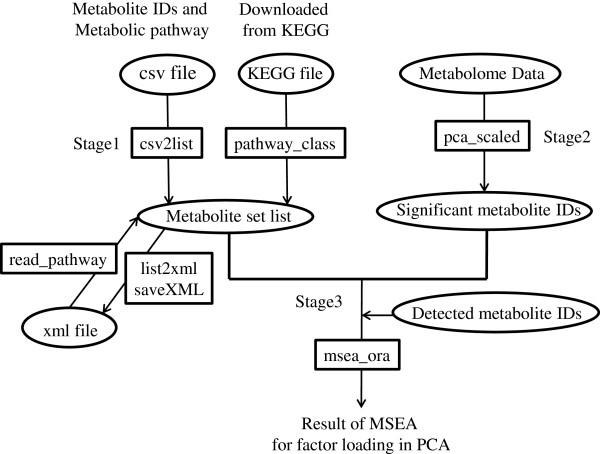
Analytical workflow for performing statistical hypothesis testing of factor loading in PCA and MSEA with the “mseapca” package.

Figure 1 show our analytical workflow used to perform the statistical hypothesis testing of the factor loading in PCA and the MSEA with the “mseapca” package. The R package “mseapca” [23] has three major features. The first creates a list of metabolic pathways. This can be generated from two file formats, csv or KEGG’s tar.gz. The csv file is used when your own metabolite set list, created by yourself, is used and KEGG’s tar.gz is used when a metabolite set originating from KEGG’s metabolic pathway is used. A csv file, in which the first column is the name of the metabolic pathway and the second column is the metabolite IDs, is manually created and converted to the list format with the “csv2list” function. A “pathway_class” function converts KEGG’s tar.gz files (e.g., hsa.tar.gz in *Homo sapiens*) to the list format of the metabolic pathway. KEGG’s tar.gz files can be downloaded from KEGG FTP, with your own license. The “mseapca” package can save a list of metabolic pathways as xml files for future reuse and feature expansion. The “list2xml” function converts the list format of the metabolic pathways to the xml format. This xml format can be saved as an xml file using the “saveXML” function in the “XML” package. The “read_pathway” function can read the created xml file and convert it to a list of metabolic pathways for the computation of MSEA.

The second feature is the “pca_scaled” function, with which to perform PCA. A data frame constructed from metabolite IDs and a metabolome data matrix is input for the “pca_scaled” function. Metabolite IDs should be matched with those in the metabolite set list. With this function, the data matrix is automatically scaled to a zero mean and unit variance (autoscaling) for each metabolite. This function can output the PC scores, factor loadings, and *p*-values and *q*-values of Benjamini and Hochberg [25], which are the results of the statistical hypothesis testing of factor loading. In this function, “factor loading” is defined as the correlation coefficient between the PC score and each metabolite level.

The third feature is the performance of MSEA. The “msea_ora” function can perform MSEA with ORA [15]. With this function, statistical hypothesis testing of the cross-tabulation is performed with the one-sided Fisher’s exact test. The “msea_sub” function performs MSEA in the same way as GSEA is implemented by Subramanian et al. [16]. Subramanian’s GSEA has two types of random permutation. In one, the class label is randomly permuted and in the other, the metabolites in the metabolite set list are randomly selected to generate the null distribution of the enrichment score. The *p*-value for the enrichment score can then be computed with both approaches. In the “msea_sub” function, the latter approach is implemented. This procedure corresponds to the “gene set” of the permutation type in the GSEA-P software [26]. A leading-edge subset analysis is also undertaken following the GSEA procedure [25].

The R package “mseapca” is freely available from the CRAN website [23]. See the reference manual for “mseapca” at the CRAN website [23] for more information.

## Results

### Case study 1: a comparative study of control and 12 h-fasted mice

We describe the use of the statistical hypothesis testing of factor loading in PCA using metabolome data from two studies. The first case study is a comparative analysis of normal and 12 h-fasted mice. Five liver samples each from the control and 12 h-fasted mice were used for the metabolomic analysis and 282 metabolites were identified.

**Figure 2:**
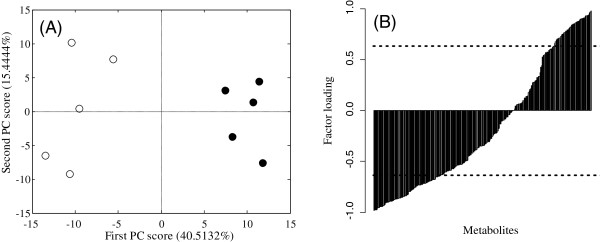
**Results of PCA in a comparative study of normal and 12 h-fasted mice. (A)** Scores plot of PC1 and PC2. Symbols: (○) control mice; (●) 12 h-fasted mice. **(B)** Factor loading plot for PC1. Metabolites are sorted in ascending order of the value for factor loading. The dotted line shows the significance level at *p* < 0.05.

A PCA of these metabolomic data was performed after they had been preprocessed by autoscaling. The scores plot of the PCA (Figure 2(A)) showed that the PC1 scores of the control and fasted mice were negative and positive, respectively. This result suggests that the PC1 score is positively related to the fasting effect. In this case, metabolites that have large positive factor loadings in PC1 tend to increase and those with negative factor loadings tend to decrease during the 12 h fast.

Statistical hypothesis testing for factor loading in PC1 was performed, and 136 metabolites were statistically significant at *p* < 0.05 (Additional file 1: Table S1). An MSEA with ORA for factor loading was performed independently for the significantly positive and negative metabolites (Table 1). Purine metabolism was significantly activated in the 12 h-fasted mice at *p* < 0.05. Glycolysis was significantly suppressed at *q* < 0.05 and the pentose phosphate pathway tricarboxylic acid (TCA) cycle, cysteine metabolism, and polyamine metabolism were significantly suppressed in the 12 h-fasted mice at *p* < 0.05. MSEA using Subramanian’s approach was also performed as a reference (Table 1). Histidine metabolism and purine metabolism had negative normalized enrichment scores (NESs), so were significantly activated in the 12 h-fasted mice at *p* < 0.05. Glycolysis had a positive NES, so was significantly suppressed at *q* < 0.05 and the pentose phosphate pathway, TCA cycle, and polyamine metabolism were significantly suppressed in the 12 h-fasted mice at *p* < 0.05. These results suggest that these two MSEA approaches are largely consistent.

**Table 1:** Results of MSEA in a comparative study of normal and 12 h-fasted mice

	**ORA**				**Subramanian's approach**		
	**Positive correlation with PC1**		**Negative correlation with PC1**		**Positive correlation with PC1**		
	**p-value**	**q-value**	**p-value**	**q-value**	**NES**	**p-value**	**q-value**
Glycolysis	1.0000	1.0000		0.0001*	0.0036**	-2.0048	0.0000*	0.0074**
Pentose phosphate pathway	1.0000	1.0000		0.0308*	0.2000	-1.6040	0.0283*	0.1781
TCA cycle	1.0000	1.0000		0.0040*	0.0519	-1.6208	0.0165*	0.2433
Glutamic acid and glutamine metabolism	1.0000	1.0000		0.4901	0.9801	-1.0936	0.3497	0.5193
Alanine, aspartic acid and asparagine metabolism	0.8254	1.0000		0.2878	0.8313	-1.1897	0.2625	0.4379
Lysine metabolism	0.8567	1.0000		0.8681	1.0000	-0.8172	0.7078	0.8274
Valine, leucine and isoleucine metabolism	1.0000	1.0000		0.7735	1.0000	-0.9392	0.5636	0.7311
Glycine, serine and threonine metabolism	0.7434	1.0000		0.0720	0.2445	-1.2557	0.1803	0.3704
Cysteine metabolism	0.6489	1.0000		0.0412*	0.2142	-1.3259	0.1611	0.3371
Methionine metabolism	0.6178	1.0000		0.8444	1.0000	0.8697	0.6147	0.7197
Shikimic acid metabolism	1.0000	1.0000		0.4901	0.9801	-1.2676	0.1745	0.3901
Histidine metabolism	0.5434	1.0000		1.0000	1.0000	1.8978	0.0080*	0.0520
Urea cycle	0.8567	1.0000		0.0507	0.2197	-1.3352	0.1524	0.3705
Proline metabolism	1.0000	1.0000		0.6001	1.0000	-1.1524	0.3041	0.4619
Polyamine metabolism	1.0000	1.0000		0.0308*	0.2000	-1.5785	0.0309*	0.1581
Tryptophan metabolism	0.7413	1.0000		0.9269	1.0000	-0.7141	0.8260	0.8764
Tyrosine metabolism	1.0000	1.0000		0.0752	0.2445	-1.3601	0.1176	0.3820
beta-alanine metabolism	0.2111	1.0000		0.8616	1.0000	0.9218	0.5531	0.7578
Taurine metabolism	1.0000	1.0000		0.3721	0.9675	-1.4114	0.1010	0.3535
Creatine metabolism	0.7874	1.0000		0.4705	0.9801	-0.8041	0.7093	0.7984
Purine metabolism	0.0285*	0.7411		0.9983	1.0000	1.6391	0.0220*	0.1290
Pyrimidine metabolism	0.9649	1.0000		0.9860	1.0000	0.7965	0.7355	0.7258
Ribonucleotide metabolism	0.3473	1.0000		1.0000	1.0000	1.1184	0.2800	0.6998
Deoxyribonucleotide	1.0000	1.0000		1.0000	1.0000	-0.6743	0.9776	0.8725
Conjugated bile acid	0.5361	1.0000		1.0000	1.0000	1.0384	0.3671	0.6834
Nicotinic acid metaboilsm	0.3473	1.0000		0.5357	0.9949	-0.8360	0.6536	0.8510

*p < 0.05, **q < 0.05.

The results of the MSEA of factor loading in PC1 suggested that the processes of energy metabolism, including glycolysis and the TCA cycle, decreased during the 12 h fast. The suppression of these metabolic pathways suggests that glycogen is drained and glucose supplementation is restricted in the mouse liver during a 12 h fast. The mean bodyweight of the normal mice was 22.20 ± 0.84 g (mean ± SD) and that of the 12 h-fasted mice was 20.0 ± 0.71 g, indicating a statistically significant reduction (*p* = 0.0021) during the 12 h fast, according to Welch’s *t* test. This result suggests that the suppression of energy metabolism results in a reduction in bodyweight.

### Case study 2: a comparative study of diabetic model mice with and without pioglitazone treatment

The *db*/*db* mouse is a model of obesity, diabetes, and dyslipidemia, in which leptin receptor activity is deficient because the mice are homozygous for a point mutation in the leptin receptor gene [27]. Pioglitazone reduces insulin resistance in the liver and reduces glucose levels in the blood [28,29]. Therefore, it is used for the treatment of diabetes.

We compared the metabolomic data for mouse liver samples from *db*/*db* mice treated with or without pioglitazone to examine the effects of administering pioglitazone to diabetic mice. Five liver samples from the untreated *db*/*db* mice and five from *db*/*db* mice administered pioglitazone were used for the metabolomic analysis and 275 metabolites were identified.

**Figure 3:**
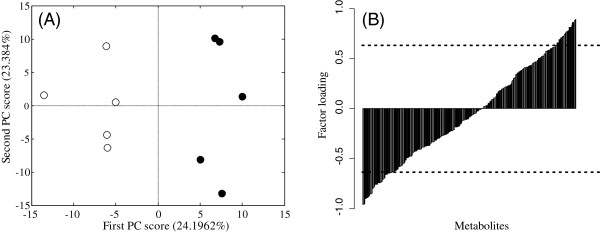
**Results of PCA in a comparative study of *db*/*db *mice treated with and without pioglitazone. (A)** Scores plot of PC1 and PC2. Symbols: (○) diabetic model mice (*db*/*db* mice) without pioglitazone; (●) *db*/*db* mice administered pioglitazone. **(B)** Factor loading plot for PC1. Metabolites are sorted in ascending order of the value for factor loading. The dotted line shows the significance level at *p* < 0.05.

We performed PCA on data preprocessed with autoscaling in a comparative study of the *db*/*db* mice treated with and without pioglitazone. The scores plot is shown in Figure 3(A). A perfect separation between the groups was achieved on the first PC axis, and we therefore focused on this axis. The PC1 scores for the *db*/*db* mice with and without pioglitazone treatment showed positive and negative values, respectively, suggesting that the PC1 score is positively related to the effect of pioglitazone.

Statistical hypothesis testing of the factor loading in PC1 was performed, and 66 metabolites were statistically significant at *p* < 0.05 (Additional file 1: Table S2). An MSEA of factor loading was performed as in the previous section (Table 2). In both MSEA with ORA and using Subramanian’s approach, glycolysis was the only statistically significant factor activated by pioglitazone at *p* < 0.05. Pioglitazone is a peroxisome proliferator-activated receptor (PPAR)-activating agent. Lee et al. [30] suggested that PPARδ ameliorates hyperglycemia by increasing the glucose flux through the regulation of gene expression. The administration of pioglitazone is known to reduce glucose levels in the blood [28,29].

**Table 2:** **Results of MSEA in a comparative study of *db*/*db *** **mice treated with and without pioglitazone**

	**ORA**				**Subramanian’s approach**		
	**Positive correlation with PC1**		**Negative correlation with PC1**		**Positive correlation with PC1**		
	**p-value**	**q-value**	**p-value**	**q-value**	**NES**	**p-value**	**q-value**
Glycolysis	0.0090*	0.2250	0.7982	1.0000	1.6888	0.0198*	0.3485
Pentose phosphate pathway	1.0000	1.0000	1.0000	1.0000	1.3813	0.1378	0.9520
TCA cycle	1.0000	1.0000	1.0000	1.0000	-1.4237	0.0916	0.8261
Glutamic acid and glutamine metabolism	1.0000	1.0000	0.5471	1.0000	-1.2740	0.2062	0.4203
Alanine, aspartic acid and asparagine metabolism	0.5531	1.0000	1.0000	1.0000	0.7161	0.8049	1.0000
Lysine metabolism	1.0000	1.0000	1.0000	1.0000	-0.6892	0.8152	0.9603
Valine, leucine and isoleucine metabolism	0.3294	1.0000	1.0000	1.0000	0.8275	0.6150	0.9834
Glycine, serine and threonine metabolism	0.1405	0.7024	0.8617	1.0000	1.1241	0.2699	0.9823
Cysteine metabolism	0.7041	1.0000	0.2461	1.0000	-0.9727	0.4840	0.8845
Methionine metabolism	1.0000	1.0000	1.0000	1.0000	1.0388	0.4167	0.9189
Shikimic acid metabolism	0.3294	1.0000	1.0000	1.0000	1.1493	0.3098	1.0000
Histidine metabolism	1.0000	1.0000	1.0000	1.0000	0.7463	0.7770	1.0000
Urea cycle	1.0000	1.0000	1.0000	1.0000	0.6208	0.9293	0.9238
Proline metabolism	0.5051	1.0000	1.0000	1.0000	0.6493	0.8869	1.0000
Polyamine metabolism	0.1344	0.7024	0.2701	1.0000	1.0695	0.3818	0.9813
Tryptophan metabolism	0.1018	0.7024	1.0000	1.0000	1.2380	0.2247	1.0000
Tyrosine metabolism	0.4521	1.0000	1.0000	1.0000	0.9319	0.5367	0.8548
beta-alanine metabolism	0.3893	1.0000	0.6321	1.0000	0.6450	0.8899	0.9596
Taurine metabolism	0.0577	0.7024	0.4410	1.0000	0.9952	0.4654	0.8048
Creatine metabolism	1.0000	1.0000	0.7206	1.0000	-0.7887	0.7230	0.9414
Purine metabolism	1.0000	1.0000	0.2583	1.0000	-1.3788	0.0947	0.5203
Pyrimidine metabolism	1.0000	1.0000	0.9252	1.0000	-0.7940	0.7196	1.0000
Ribonucleotide metabolism	1.0000	1.0000	0.2461	1.0000	-1.3605	0.1215	0.3792
Conjugated bile acid	1.0000	1.0000	0.4687	1.0000	-0.5472	0.9689	0.9709
Nicotinic acid metaboilsm	0.3161	1.0000	0.5431	1.0000	0.9991	0.4427	0.8973

*p < 0.05.

In the present study, the glucose blood level was 369.6 ± 64.8 mg/dL (mean ± SD) in the *db*/*db* mice and 332.8 ± 131.9 mg/dL in the *db*/*db* mice administered pioglitazone. The reduction in blood glucose was not significant (*p* = 0.596) after the administration of pioglitazone, according to Welch’s *t* test. This result suggests that a metabolomic analysis can detect subtle changes in the glycolysis pathway caused by the administration of pioglitazone, although confirmatory experiments (e.g., evaluating the expression levels of PPARα) might be required to confirm our biological inferences.

## Discussion

Metabolite selection by statistical hypothesis testing of the factor loading in PCA has several advantages. This approach was applied to metabolomic data in two case studies of mouse liver samples. In the first case study, 136 of 282 metabolites correlated significantly with the PC1 score associated with the groups, and in the second study, 66 of 275 metabolites showed such a correlation. Thus, the number of significant metabolites was two-fold higher in the first case study than in the second case study. This suggests that to set a previously determined number of metabolites (e.g., the top 10 metabolites) is inappropriate because the number of significant metabolites differs in each study. We also note the relationship between the contribution ratio and the number of significant metabolites for factor loading in PCA. The ratio of the number of significant metabolites to all the detected metabolites was 0.482 (= 136/282) in the first case study and 0.24 (= 66/275) in the second case study. The contribution ratio in PC1 was 40.5% in the first case study and 24.2% in the second case study. This result suggests that an implicit relationship exists between the contribution ratio and the number of significant metabolites in samples of the same size.

In both case studies, we focused on PC1 (Figure 2 and Figure 3), which differed between the groups. We then compared this approach with ordinary statistical hypothesis testing, such as with a *t* test. According to Welch’s *t* test, 122 metabolites were significant in the first case study and 56 metabolites were significant in the second case study. When we compared the metabolites selected with Welch’s *t* test and those selected with the statistical test of factor loading, 112 metabolites and 47 metabolites in case studies 1 and 2, respectively, were common to both studies. Most significant metabolites were selected with both approaches. This fact suggests that the statistical testing of factor loading in PCA can be readily used to select metabolites as a special case of the two-sample test when the difference between the groups appears in the PC score. The result of MSEA for statistically significant metabolites with positive *t* statistics on Welch’s *t* test showed that purine metabolism was statistically significant at *p* < 0.05, and the negative *t* statistics showed that glycolysis and the pentose phosphate pathway were statistically significant at *p* < 0.05 (Additional file 1: Table S3). In a positive case, the statistically significant metabolic pathway identified by MSEA was consistent with both approaches. In a negative case, the statistically significant metabolic pathway was partly consistent, but the number of statistically significant metabolic pathways was fewer with Welch’s *t* test than with the statistical hypothesis testing of factor loading in PCA. These results depend on the result that the number of significant metabolites was almost same with Welch’s *t* test (51 metabolites) and with statistical hypothesis testing of factor loading in PCA (49 metabolites) in positive cases, but was fewer with Welch’s *t* test (71 metabolites) than with the statistical hypothesis testing of factor loading in PCA (87 metabolites) in negative cases.

In metabolomics, complex studies (e.g., the fermentation process by a microorganism [31]) can involve various time points or groups, or the administration of drugs at various concentrations under various conditions [32]. In these complex studies, a statistical method for testing should be selected from various methods, such as analysis of variance and multiple comparison procedures, depending on the situation. In our analytical workflow of PCA, a specific PC score was selected and we simply performed the statistical hypothesis testing for factor loading corresponding to this selected PC under any circumstances. The statistical testing of factor loading in PCA can be widely used, not only in two-sample studies but also in various studies when an association between the PC score and the phenotype can be found.

MSEA was performed for significant metabolites and acceptable biological inferences were drawn in the two case studies. With the conventional approach, a previously determined number of metabolites (e.g., 10 metabolites) from which to draw biological inferences is subjectively selected. Using this approach, MSEA was performed for the top 10 metabolites with large negative factor loadings in the first case study. No significant metabolic pathway was detected at *p* < 0.05 (data not shown). In this case, 10 metabolites was too small a sample from which to draw acceptable biological inferences. Even if significant metabolic pathways are detected when MSEA is applied to insignificant metabolites, it is doubtful whether these metabolic pathways are statistically or biologically meaningful. To draw unbiased biological inferences using a statistical analysis, significant metabolites must be selected with statistical tests of factor loading when using PCA.

In this study, two MSEA methods were used, with either ORA or Subramanian’s approach. As a way of using factor loading for GSEA, Fehrmann et al. [33] designated the PC score associated with phenotype as the “transcriptional system regulator” (TSR) score, and factor loading corresponding to the TSR score is used for GSEA with Subramanian’s approach. This method directly uses factor loading, but does not use the results of statistical hypothesis testing of factor loading. As far as we know, an approach combining GSEA or MSEA with the results of statistical hypothesis testing of factor loading in PCA has not been reported until now.

The results of both MSEA with ORA or Subramanian’s approach produced almost the same results in our two case studies. In a comparison of the computational time required by the two MSEA approaches, the first case study required 441.43 seconds using Subramanian’s approach and 0.83 seconds with ORA. This result shows that MSEA with ORA has the advantage of lower computational cost. Conventionally, PCA and MSEA can be computed independently in different steps or with different software. There has been no software that can compute the sequence from PCA and statistical hypothesis testing of factor loading to MSEA. Therefore, we developed the R package “mseapca” to compute the whole sequence from the statistical hypothesis testing of factor loading in PCA to MSEA.

## Conclusions

In metabolomics, the targeted metabolites from which biological inferences are drawn are selected subjectively when factor loading is used in PCA. We have proposed a statistical procedure to select metabolites using the statistical hypothesis testing of factor loading in PCA. These significant metabolites are then used to identify significant metabolic pathways with MSEA. We applied this approach to two metabolomic datasets from mouse liver samples, with acceptable results in terms of previous biological knowledge. We developed an R package “mseapca” to allow the ready use of our approach. Many researchers use PCA in metabolomics. Our approach can improve the existing use of PCA in this field and is expected to be widely applicable to other omics data, including gene expression and proteomic data.

## Abbreviations

CE–MS: Capillary electrophoresis–mass spectrometry; PCA: Principal component analysis; MSEA: Metabolite set enrichment analysis; GSEA: Gene set enrichment analysis; GO: Gene ontology; ORA: Overrepresentation analysis; KEGG: Kyoto Encyclopedia of Genes and Genomes; TCA: Tricarboxylic acid; NESs: Normalized enrichment scores; TSR: Transcriptional system regulator.

## Competing interests

The authors declare that they have no competing interests.

## Authors’ contributions

HY proposed the method, performed the statistical analysis, developed the software package, and wrote the manuscript. TF edited the list of metabolic pathways. HS performed the metabolomic analysis and processed the analytical data. YO supervised all research experiments. TF, HS, GI, KK, and YO interpreted the metabolomic data biologically. All authors have read and approved the final manuscript.

## Supplementary Material

Additional file 1: Table S1 and S2 Results of statistical hypothesis testing of factor loading in PC1 and Welch’s *t* test and MSEA for the two case studies. **Table S3.** Result of MSEA using ORA for significant metabolites selected by Welch's *t*-test in a comparative study of normal and 12 h-fasted mice. Click here for file

## References

[B1] Lavine B Workman J Chemometrics Anal Chem 2010 82 12 4699 4711 10.1021/ac101202z 20481510

[B2] Jolliffe IT Principal component analysis 2002 2 New York: Springer-Verlag

[B3] Barker M Rayens W Partial least squares for discrimination J Chemometr 2003 17 3 166 173 10.1002/cem.785

[B4] Yamamoto H Yamaji H Fukusaki E Ohno H Fukuda H Canonical correlation analysis for multivariate regression and its application to metabolic fingerprinting Biochem Eng J 2007 40 199 204

[B5] Ringnér M What is principal component analysis? Nat Biotechnol 2008 26 303 304 10.1038/nbt0308-303 18327243

[B6] Landgrebe J Wurst W Welzl G Permutation-validated principal components analysis of microarray data Genome Biol 2002 3 4 1 11 10.1186/gb-2002-3-4-research0019 PMC115254 11983060

[B7] Dileo MV Strahan GD den Bakker M Hoekenga OA Weighted correlation network analysis (WGCNA) applied to the tomato fruit metabolome PLoS One 2011 6 10 e26683 10.1371/journal.pone.0026683 22039529 PMC3198806

[B8] Dewar BJ Keshari K Jeffries R Dzeja P Graves LM Macdonald JM Metabolic assessment of a novel chronic myelogenous leukemic cell line and an imatinib resistant subline by H NMR spectroscopy Metabolomics 2010 6 3 439 450 10.1007/s11306-010-0204-0 20676217 PMC2899017

[B9] Maruyama K Takeda M Kidokoro S Yamada K Sakuma Y Urano K Fujita M Yoshiwara K Matsukura S Morishita Y Sasaki R Suzuki H Saito K Shibata D Shinozaki K Yamaguchi-Shinozaki K Metabolic pathways involved in cold acclimation identified by integrated analysis of metabolites and transcripts regulated by DREB1A and DREB2A Plant Physiol 2009 150 4 1972 1980 10.1104/pp.109.135327 19502356 PMC2719109

[B10] Van den Berg RA Hoefsloot HC Westerhuis JA Smilde AK van der Werf MJ Centering, scaling, and transformations: improving the biological information content of metabolomics data BMC Genomics 2006 7 142 10.1186/1471-2164-7-142 16762068 PMC1534033

[B11] Afifi A May S Clark VA Practical Multivariate Analysis 2011 5 London: Chapman and Hall/CRC 364 366

[B12] Pedro RP Donald AJ Keith MS Giving meaningful interpretation to ordination axes: assessing loading significance in principal component analysis Ecology 2003 84 2347 2363 10.1890/00-0634

[B13] Xia J Wishart DS MSEA: a web-based tool to identify biologically meaningful patterns in quantitative metabolomic data Nucleic Acids Res 2010 38 Web Server issue W71 W77 20457745 10.1093/nar/gkq329 PMC2896187

[B14] Xia J Wishart DS Web-based inference of biological patterns, functions and pathways from metabolomic data using MetaboAnalyst Nat Protoc 2011 6 743 760.15 10.1038/nprot.2011.319 21637195

[B15] Draghici S Khatri P Martins RP Ostermeier GC Krawetz SA Global function profiling of gene expression Genomics 2003 81 98 104 10.1016/S0888-7543(02)00021-6 12620386

[B16] Subramanian A Tamayo P Mootha VK Mukherjee S Ebert BL Gillette MA Paulovich A Pomeroy SL Golub TR Lander ES Mesirov JP Gene set enrichment analysis: a knowledge-based approach for interpreting genome-wide expression profiles Proc Natl Acad Sci U S A 2005 102 43 15545 15550 10.1073/pnas.0506580102 16199517 PMC1239896

[B17] Goeman JJ van de Geer SA de Kort F van Houwelingen HC A global test for groups of genes: testing association with a clinical outcome Bioinformatics 2004 20 1 93 99 10.1093/bioinformatics/btg382 14693814

[B18] Ooga T Sato H Nagashima A Sasaki K Tomita M Soga T Ohashi Y Metabolomic anatomy of an animal model revealing homeostatic imbalances in dyslipidaemia Mol Biosyst 2011 7 4 1217 1223 10.1039/c0mb00141d 21258713

[B19] Soga T Heiger DN Amino acid analysis by capillary electrophoresis electrospray ionization mass spectrometry Anal Chem 2000 72 1236 1241 10.1021/ac990976y 10740865

[B20] Soga T Ueno Y Naraoka H Ohashi Y Tomita M Nishioka T Analysis of nucleotides by pressure-assisted capillary electrophoresis mass spectrometry using silanol mask technique J Chromatogr A 2007 1159 125 133 10.1016/j.chroma.2007.05.054 17543971

[B21] Sugimoto M Wong D Hirayama A Soga T Tomita M Capillary electrophoresis mass spectrometry-based saliva metabolomics identifies oral, breast and pancreatic cancer-specific profiles Metabolomics 2010 6 78 95 10.1007/s11306-009-0178-y 20300169 PMC2818837

[B22] R Development Core Team R: A language and environment for statistical computing 2005 Vienna, Austria: R Foundation for Statistical Computing

[B23] mseapca: Metabolite set enrichment analysis for factor loading in principal component analysis http://cran.r-project.org/web/packages/mseapca/10.1186/1471-2105-15-51 PMC4015128 24555693

[B24] Kanehisa M Goto S KEGG: Kyoto encyclopedia of genes and genomes Nucleic Acids Res 2000 28 27 30 10.1093/nar/28.1.27 10592173 PMC102409

[B25] Benjamini Y Hochberg Y Controlling the false discovery rate: a practical and powerful approach to multiple testing J R Stat Soc Ser B 1995 57 1 289 300

[B26] Subramanian A Kuehn H Gould J Tamayo P Mesirov JP GSEA-P: a desktop application for gene Set enrichment analysis Bioinformatics 2007 23 23 3251 3253 10.1093/bioinformatics/btm369 17644558

[B27] Sharma K McCue P Dunn SR Diabetic kidney disease in the db/db mouse Am J Physiol Renal Physiol 2003 284 F1138 F1144 12736165 10.1152/ajprenal.00315.2002

[B28] Kemnitz JW Elson DF Roecker EB Baum ST Bergman RN Maglasson MD Pioglitazone increases insulin sensitivity, reduces blood glucose, insulin, and lipid levels, and lowers blood pressure, in obese, insulin-resistant rhesus monkeys Diabetes 1994 43 204 211 10.2337/diab.43.2.204 8288044

[B29] Smith U Pioglitazone: mechanism of action Int J Clin Pract 2001 121 13 18 11594239

[B30] Lee CH Olson P Hevener A Mehl I Chong LW Olefsky JM Gonzalez FJ Ham J Kang H Peters JM Evans RM PPARδ regulates glucose metabolism and insulin sensitivity Proc Natl Acad Sci U S A 2006 103 9 3444 3449 10.1073/pnas.0511253103 16492734 PMC1413918

[B31] Yamamoto H Yamaji H Abe Y Harada K Waluyo D Fukusaki E Kondo A Ohno H Fukuda H Dimensionality reduction for metabolome data using PCA, PLS, OPLS, and RFDA with differential penalties to latent variables Chemom Intell Lab Syst 2009 98 2 136 142 10.1016/j.chemolab.2009.05.006

[B32] Timmerman ME van der Greef J Lamers RAN Huub C Hoefsloot J Smilde AK Jansen JJ ANOVA-simultaneous component analysis (ASCA): a new tool for analyzing designed metabolomics data Bioinformatics 2005 21 13 3043 3048 10.1093/bioinformatics/bti476 15890747

[B33] Fehrmann RSN de Jonge HJM ter Elst A de Vries A Crijns AGP Weidenaar AC Gerbens F de Jong S van der Zee AGJ de Vries EGE Kamps WA Hofstra RMW te Meerman GJ de Bont ESJM A New perspective on transcriptional system regulation (TSR): towards TSR profiling PLoS One 2008 3 2 e1656 10.1371/journal.pone.0001656 18297136 PMC2250855

